# Reverse Engineering of Ewing Sarcoma Regulatory Network Uncovers PAX7 and RUNX3 as Master Regulators Associated with Good Prognosis

**DOI:** 10.3390/cancers13081860

**Published:** 2021-04-13

**Authors:** Marcel da Câmara Ribeiro-Dantas, Danilo Oliveira Imparato, Matheus Gibeke Siqueira Dalmolin, Caroline Brunetto de Farias, André Tesainer Brunetto, Mariane da Cunha Jaeger, Rafael Roesler, Marialva Sinigaglia, Rodrigo Juliani Siqueira Dalmolin

**Affiliations:** 1 Bioinformatics Multidisciplinary Environment—IMD, Federal University of Rio Grande do Norte, Natal 59078-400, Brazil; marcel.ribeiro-dantas@curie.fr (M.d.C.R.-D.); xdanilo@ufrn.edu.br (D.O.I.); 2 Laboratoire Physico Chimie Curie, UMR168, Institut Curie, Université PSL, Sorbonne Université, 75005 Paris, France; 3 Children’s Cancer Institute, Porto Alegre 90620-110, Brazil; matheusdalmolin@ufrn.edu.br (M.G.S.D.); labpesquisa@ici.ong (C.B.d.F.); andrebrunetto@ici.ong (A.T.B.); labpesquisa1@ici.ong (M.d.C.J.); msinigaglia@ici.ong (M.S.); 4 Cancer and Neurobiology Laboratory, Experimental Research Center, Clinical Hospital (CPE HCPA), Porto Alegre 90035-903, Brazil; rafaelroesler@hcpa.edu.br; 5 Department of Pharmacology, Institute for Basic Health Sciences, Federal University of Rio Grande do Sul, Porto Alegre 90050-170, Brazil; 6 Department of Biochemistry—CB, Federal University of Rio Grande do Norte, Natal 59078-970, Brazil

**Keywords:** pediatric cancer, transcription factor, systems biology, cancer of unknown primary, regulome

## Abstract

**Simple Summary:**

Ewing Sarcoma is a rare cancer that, when localized, has an overall five-year survival rate of 70%. Patients with metastasis have a five-year survival rate of 15 to 30%. Early analysis of patient prognosis can be crucial to provide adequate treatment and increase chances of survival. Besides, it is a disease with several gaps in our understanding, including regulation of genes and which transcription factors are master regulators. This work addresses these two topics by inferring gene regulatory networks that allow us to identify putative master regulators to predict patient prognosis. We were able to identify two sets of master regulators that can predict good and bad patient outcomes.

**Abstract:**

Ewing Sarcoma (ES) is a rare malignant tumor occurring most frequently in adolescents and young adults. The ES hallmark is a chromosomal translocation between the chromosomes 11 and 22 that results in an aberrant transcription factor (TF) through the fusion of genes from the FET and ETS families, commonly EWSR1 and FLI1. The regulatory mechanisms behind the ES transcriptional alterations remain poorly understood. Here, we reconstruct the ES regulatory network using public available transcriptional data. Seven TFs were identified as potential MRs and clustered into two groups: one composed by PAX7 and RUNX3, and another composed by ARNT2, CREB3L1, GLI3, MEF2C, and PBX3. The MRs within each cluster act as reciprocal agonists regarding the regulation of shared genes, regulon activity, and implications in clinical outcome, while the clusters counteract each other. The regulons of all the seven MRs were differentially methylated. PAX7 and RUNX3 regulon activity were associated with good prognosis while ARNT2, CREB3L1, GLI3, and PBX3 were associated with bad prognosis. PAX7 and RUNX3 appear as highly expressed in ES biopsies and ES cell lines. This work contributes to the understanding of the ES regulome, identifying candidate MRs, analyzing their methilome and pointing to potential prognostic factors.

## 1. Introduction

Ewing Sarcoma (ES) is an aggressive bone and soft tissue sarcoma, occurring most frequently in adolescents and young adults, of which the hallmark is a chromosomal translocation between chromosomes 11 and 22, involving genes from the FET and the ETS transcription factor families. Bone is the most frequent site of origin, but about 15% to 20% of Ewing sarcoma emerges in bone-associated soft tissue [1,2,3]. EWSR1-FLI1 has been reported as the most frequent fusion in ES, coding for a chimeric protein that functions as an aberrant transcription factor [4]. The EWSR1-FLI1 chimeric protein is known to alter the chromatin leading to mistargeting, dysregulation of chromatin state, and eventually transcriptional impairing [5,6]. The impact of EWSR-FLI1 fusion on ES transcriptome is still poorly understood, and the regulatory mechanisms behind those transcriptional alterations have not yet been deeply investigated.

ES was first described as a presumed diffuse endothelioma of bone [7]. The key ES prognostic factor is the presence of detectable metastasis at diagnosis, and patients with bony metastases (with or without lung involvement) have a very poor prognosis, mostly when compared to patients with exclusively lung metastasis [8]. It is known that the chimeric protein alters the transcriptional pattern, and some of ES transcriptional abnormalities have been associated with epigenetic modifications induced by EWSR1-FLI1 [4]. There is no ES cell of origin identified so far, and several ES tissues of origin were proposed, including endothelial, vascular pericytes or smooth muscle, primitive vascular mesenchyme, pluripotential uncommitted mesenchyme, osteoblastic (based on collagen matrix synthesis patterns) and small cell osteosarcoma, and/or mesenchymal chondrosarcoma [2,9]. In recent decades, studies converged on two putative ES cells of origin: human mesenchymal stem cells (hMSC) and human neural crest cells (hNCC) [4]. There are also studies that point towards a neuro-mesenchymal stem cell phenotype [10].

Gene expression is regulated by the activity of regulatory molecules such as transcription factors. It is well described that a small number of transcription factors, which acts as master regulators (MRs), might handle the cell fate in different cellular models [11,12]. The reconstruction of regulatory networks based on transcriptional information has been successfully applied to different types of cancer, including breast cancer, pancreatic ductal adenocarcinoma, and neuroblastoma [13,14,15,16]. The regulatory network reconstruction aims to identify those transcription factors at the top of the transcriptional regulatory hierarchy, the MRs [17,18]. Identifying such MRs could help predict patient prognostic and pointing out putative biomarkers that could lead to better diagnosis and treatment protocols. Compared with other solid cancers, and cancers that have recurrent mutations, the number of recurrent somatic mutations in ES is limited [19,20,21,22]. Therefore, ES development is known as a transcription-related phenomenon, which points to regulatory analysis as a promising strategy in ES investigation. Here, we have inferred the ES regulatory network based on transcriptional data available in public databases. We also identified a set of seven putative transcription factors which acts as master regulators in ES and analyzed their methylation profile. Those master regulators clustered in two groups with antagonistic behavior between the groups regarding the regulation of shared genes, regulon activity, and implications in clinical outcome.

## 2. Materials and Methods

### 2.1. Data Acquisition and Processing

Data used to infer the ES regulatory networks were obtained from Gene Expression Omnibus (GEO) [23] (accession numbers GSE34620 and GSE63157). The dataset GSE34620 comprises expression data (Affymetrix Human Genome U133 Plus 2.0 Array) from 117 ES biopsies [24] and the dataset GSE63157 comprises expression data (Affymetrix Human Exon 1.0 ST Array) from 85 ES biopsies [25]. Data used to infer ES gene signature was also obtained from GEO (accession numbers GSE73610 and GSE67073) [26,27]. The dataset GSE73610 comprises expression data (RNA-Seq profiling Illumina Genome Analyzer IIx, Homo sapiens) from 3 ES cell lines and 2 hMSC cell lines and the dataset GSE67073 comprises expression data (RNA-Seq profiling Illumina HiSeq 2000, Homo sapiens) from four induced pluripotent stem cells (iPSC)-derived neural crest populations from familial dysautonomia patients and 2 iPSC-derived neural crest populations from healthy volunteers. Data used for survival analysis was obtained from GSE63157 (also used for Ewing Sarcoma regulatory network inference) and from GSE17618, both retrieved from GEO. The dataset GSE17618 comprises expression data (Affymetrix Human Genome U133 Plus 2.0 Array) from 44 ES biopsies and 11 ES cell line samples [27].

To assess the gene expression of the master regulators in different tissues and solid pediatric cancers, we used once again the dataset GSE34620 for ES, in addition to the following: GSE16476, comprising expression data (Affymetrix Human Genome U133 Plus 2.0 Array) from 88 Neuroblastoma biopsies; GSE53224 comprising expression data (Affymetrix Human Genome U133 Plus 2.0 Array) from 53 biopsies of Wilm’s tumor; GSE75271 comprising expression data (Affymetrix Human Genome U133 Plus 2.0 Array) from 55 biopsies of hepatoblastoma, GSE87437 comprising expression data (Affymetrix Human Genome U133 Plus 2.0 Array) from 21 biopsies of high-grade osteosarcoma, GSE29684 comprising expression data (Affymetrix Human Genome U133 Plus 2.0 Array) from 20 biopsies of retinoblastoma, GSE66533 comprising expression data (Affymetrix Human Genome U133 Plus 2.0 Array) from 58 biopsies of rhabdomyosarcoma and GSE3526 comprising expression data (Affymetrix Human Genome U133 Plus 2.0 Array) from 353 samples of 65 different healthy tissues. The criterion chosen for this analysis was the most common solid pediatric tumors, microarray platform type, and the number of samples.

Microarray raw data (GSE34620 and GSE17618, Affymetrix Human Genome U133 Plus 2.0 Array) normalization (Robust Multichip Average, RMA method) and quality control was performed with the Affy Bioconductor/R package [28]. For GSE63157 (Affymetrix Human Exon 1.0 ST Array) normalization (Robust Multichip Average, RMA method) and quality control was performed with oligo Bioconductor/R package [29]. Annotation data for Affymetrix Human Genome U133 Plus 2.0 Array was obtained from hgu133plus2.db and for Affymetrix Human Exon 1.0 ST Array from huex10sttranscriptcluster.db R packages. RNA-seq data were processed according to the Tuxedo protocol. Briefly, we used fastqdump to convert SRA compressed files into FASTQ format files and fastqc to assess the quality of them. Then we run TopHat to align reads to the hg19 reference transcriptome. For the comparison of gene expression of the master regulators among solid pediatric tumors and healthy tissues, the datasets GSE34620, GSE16476, GSE53224, GSE75271, GSE87437, GSE29684, GSE66533, and GSE3526 were normalized together. Looking for samples on the same platform type was important here since different microarray platforms have a different set of probes.

In this work, we analyzed three datasets with ES biopsies. For GSE63157, data was from biopsies collected from patients on Children’s Oncology Group (COG) and EuroEwing. Molecular analysis of COG and EuroEwing tumors was performed using RT-PCR for EWS-FLI1 and EWS-ERG fusions. Forty-six samples obtained from the COG Biorepository in Columbus, Ohio (Cooperative Human Tissue Network - CHTN) were prospectively acquired from patients on clinical trials INT-0154 (CCG-7942, POG-9354) and AEWS0031, the two most recent COG clinical protocols for patients with localized Ewing sarcoma (ES). An independent set of 39 tumor samples was obtained from the EuroEwing tumor biorepository in Muenster, Germany and were prospectively acquired from patients registered on EICESS 92 (European Intergroup Cooperative Ewing’s Sarcoma Study) and EuroEwing 99.46 out of the 85 samples have information about the primary tumor site (Under three categories: other, extremity and pelvis) [25]. For GSE17618, the patient material for study was taken prior to any treatment in 29 cases, and in 15 cases, chemotherapeutics, radiation therapy, and/or surgical treatment was applied before material was collected [30]. In the case of GSE34620, samples were from the CIT program (Cartes d’Identité des Tumeurs research program) from the french Ligue Nationale Contre le Cancer. The published dataset article details that all cases included in the study showed a specific EWSR1-ETS fusion and the molecular diagnosis was performed in Institut Curie [24].

### 2.2. Regulatory Network Inference and Master Regulator Analysis

**Figure A1:**
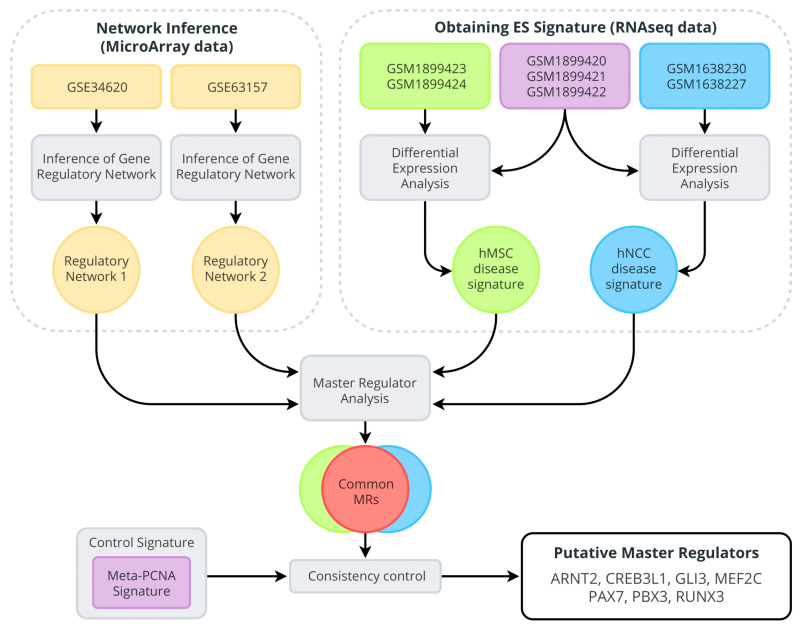
Master regulator analysis flowchart. Human mesenchymal stem cells (hMSC) and human neural crest cells (hNCC) were used along with ES cell lines to obtain the ES signatures. One analysis using hMSC as control and another using hNCC as control. Gene expression data from GSE34620 (N = 117) and GSE63157 (N = 85) were used to infer the regulatory networks 1 and 2, respectively. The networks were interrogated with the two gene signatures (disease signature with hMSC as control and disease signature with hNCC as control), and a set of shared master regulators were identified: ARNT2, CREB3L1, GLI3, MEF2C, PAX7, PBX3, and RUNX3. The meta-PCNA signature was used to filter for transcription factors commonly associated with cancer development. However, none from the ES MRs were filtered.

The regulatory network inferences and the master regulator analyses were performed using RTN (Reconstruction of Transcriptional Networks), a Bioconductor/R package that provides several tools for the reconstruction and analyses of transcriptional networks, such as the Algorithm for the Reconstruction of Accurate Cellular Networks (ARACNe), and the Master Regulator Analysis (MRA) algorithms [15]. Briefly, the RTN algorithm measures statistical dependence of gene expression data along with a previously defined set of transcription factors (TFs) to infer a TF-centric regulatory network. The Transcriptional Network Algorithm (TNI) computes the association between a transcription factor and each potential regulated gene, removing spurious associations by permutation analysis (BH adjusted *p*-value 0.05). We have inferred two ES regulatory networks by using two datasets containing ES expression data from 117 biopsies (GSE34620), used to infer Network 1, and 85 biopsies (GSE63157), used to infer Network 2, and a list of 1388 human transcription factors available in Fletcher2013b Bioconductor/R package [15]. The MRA looks for regulatory units (regulons) that are enriched for a gene signature (BH adjusted *p*-value 0.05), pointing out putative transcription factors that are relevant to the disease, known as Master Regulators (MRs). Here, we obtained two ES signatures: one by performing a differential expression analysis comparing hMSC (GSE73610 *N* = 2) against ES cell lines (GSE73610 *N* = 3) and another by performing a differential expression analysis comparing hNCC (GSE67073 *N* = 2) against the same ES cell line samples (GSE73610 *N* = 3). RNAseq samples were processed with the Tuxedo protocol, and Cuffdiff was used to quantify gene expression in each condition and test for differential expression. Genes with adjusted *p*-value lesser or equal than 0.05 were considered to be differentially expressed. We used both signatures to perform the MRA pipeline to network 1 and network 2 (Figure A1).

### 2.3. Differential Methylation Analysis

The differential methylation analysis was performed comparing 15 samples from different ES cell lines with 9 samples from hMSC cell lines. These 9 samples from hMSC are divided between 6 samples from patients with ES and 3 from healthy donors. The complete study consists of 24 samples (GSE118872) [31]. We used the methylationArrayAnalysis package from Bioconductor [32], and followed their pipeline instructions. Data normalization was performed using the Genome Studio, the standard software provided by Illumina, through the function preprocessIllumina.

Quality control was performed looking for failed positions (this is defined as both the methylated and unmethylated channel reporting background signal levels), as recommended in the pipeline manual, and with a *p*-value threshold of 0.01. At the end, no sample was required to be filtered out. After that, probe-wise differential methylation analysis was performed (Bonferroni adjusted *p*-value < 0.01). We then used Fisher’s Exact Test (Bonferroni adjusted *p*-value < 0.01) to check if the regulons of the putative master regulators inferred in the Master Regulator Analysis were significantly enriched with genes that were found to be differentially methylated in the probe-wise differential methylation analysis. This enrichment analysis was performed 2 times: (a) with regulons inferred in Network 1, and (b) with regulons inferred in Network 2.

### 2.4. Gene Ontology Functional Enrichment

To investigate the functional enrichment of genes regulated by each of the master regulators, we merged the regulons of each of the master regulators from Network 1 and 2. The Gene Ontology functional enrichment was performed using the clusterProfiler R Package aimed at the Biological Processes ontology [33]. The adjusted *p*-value < 0.05 (Benjamini-Hochberg correction) was considered to be significant.

### 2.5. Network Visualization

We used the RedeR Bioconductor/R package [34] to visualize two types of networks generated by the RTN package: association maps and tree-and-leaf representations. In association maps, nodes in the network are transcription factors, and the edge width between any two nodes is related to the number of genes mutually regulated by each pair of transcription factors, i.e., the edge width refers to the regulatory overlap between regulons. Tree-and-leaf representation is similar to the association map, with the exception that edge width is fixed and nodes are hierarchically organized throughout the network according to the overlap of regulated genes. The regulon clusters are formed according to the overlap of genes regulated by these transcription factors. Regulons with less than 15 genes are not represented in the networks, a default cut-off in the RTN package.

### 2.6. Master Regulators Activity

We used the RTN package to measure MR regulatory activity, i.e., the enrichment score of the regulatory activity of each regulon compared to the other regulons in the set for every patient. The RTN package measures for every patient how the expression of every gene deviates towards the average expression of that gene for all patients in the cohort and then applies the Two-Tailed Gene Set Enrichment Score analysis (GSEA2). Briefly, gene expression was first converted into z-score and, in each sample, all genes were sorted by the z-score and then used as the reference list in GSEA2. The TFs activity level was approximated by the Normalized Enrichment Score (NES) computed for its regulon. The results of the two analyses were plotted as a heatmap along with dendrograms.

### 2.7. Survival Analysis

From the ES datasets used in this study, only GSE63157 (N = 85) and GSE17618 (N = 44) had survival data. The survival R package was used to analyze patient overall survival in terms of the activity of the regulon of each putative master regulator. Samples were divided into two groups based on the median of the regulatory activity of each regulon. The regulons with activity values above the median were classified as “high regulon activity” and values below the median as “low regulon activity”. The median was calculated for every regulon of each patient. The *p*-values for the comparison of the survival curves in the Kaplan-Meier estimator were calculated with the log-rank test.

For the GSE17618 cohort, with too few samples to perform network inference, we used the regulon structure of the cohort one (the cohort with the largest sample size used for network inference, *N* = 117), i.e., which genes were associated with each putative master regulator, but with the expression values of the GSE17618 cohort.

### 2.8. RT-qPCR Analysis

For the in vitro expression gene analysis of master regulators identified in silico, we used different representative cell lines of Ewing Sarcoma (RD-ES and SK-ES), Neuroblastoma (SH-SY5Y and SK-N-Be(2)), Hepatoblastoma (HepG2) and Medulloblastoma (Daoy and D283). The total RNA extraction was performed with SV Total RNA Isolation System kit (Promega, Madison, USA) according to manufacturer’s instructions and quantified in Nanodrop (Thermo Fisher Scientific, Waltham, USA). The cDNA was obtained using the GoScript Reverse System (Promega) according to manufacturer’s instructions. The qPCR of the master regulators cDNA (PAX7, RUNX3, ARNT2, CREB3L1, GLI3, MEF2C, PBX3) were amplified using PowerUp SYBR Green Master Mix (Thermo Fisher Scientific.) and the relative gene expression was analysed using 2${}^{\left(-DDCt\right)}$ method [35]. The RD-ES was used as control and ACTB was used as internal control. Statistical analysis was performed by one-way analysis of variance (ANOVA) followed by Bonferroni post-hoc test; *p* values under 0.05 were considered to indicate statistical significance.

## 3. Results

### 3.1. Regulatory Network Inference and Master Regulator Analysis

**Figure 1:**
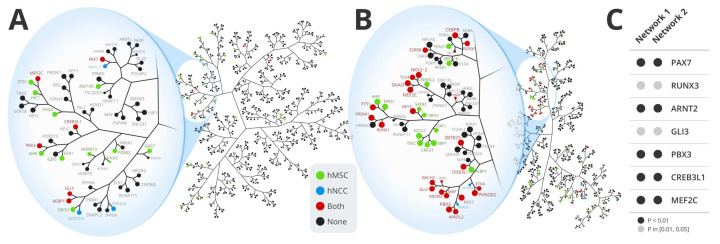
Tree-and-leaf representation of Ewing Sarcoma regulatory networks and methylation profile. Regulatory networks 1 (**A**) and 2 (**B**) inferred from ES datasets GSE34620 and GSE63157, respectively. Nodes represent regulatory units (regulons) labeled by their transcription factors, and edges represent their relationship as the overlap of mutually regulated genes. Network nodes are colored according to the master regulator analysis (MRA) results, carried out with the disease signatures obtained with hMSC and hNCC as ES cells of origin. Methylation profile (**C**) of the seven regulons composing the intersection of both networks (networks 1 and 2), and both signatures (hMSC and hNCC). The color code in C represents methylation *p*-value as indicated.

**Figure A2:**
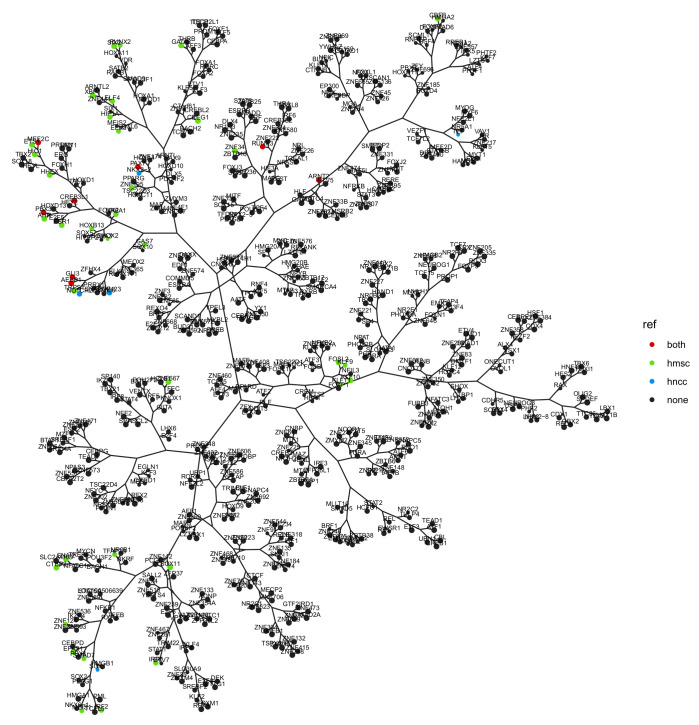
Tree-and-leaf representation of network 1 (GSE34620). Nodes are representation of regulons, labeled by the transcription factor that regulates them and colored according to its classification as master regulator for the hMSC signature (light green), hNCC signature (light blue), both signatures (red) and not a master regulator (black). The edges are the result of a hierarchical analysis on regulon overlap, i.e., the more regulated genes two regulons have in common, the closer they are in the representation.

**Figure A3:**
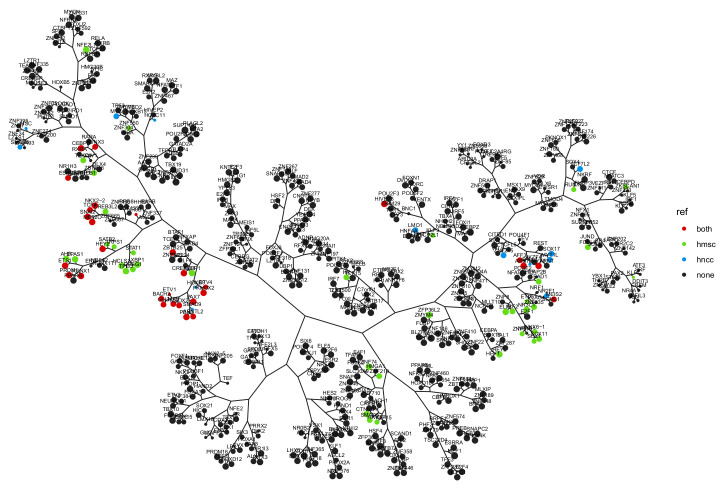
Tree-and-leaf representation of network 2 (GSE63157). Nodes are representation of regulons, labeled by the transcription factor that regulates them and colored according to its classification as master regulator for the hMSC signature (light green), hNCC signature (light blue), both signatures (red) and not a master regulator (black). The edges are the result of a hierarchical analysis on regulon overlap, i.e., the more regulated genes two regulons have in common, the closer they are in the representation.

We have inferred ES regulatory networks based on two cohorts (GSE34620 and GSE63157) containing transcriptional data from 117 patients (regulatory network 1) and 85 patients (regulatory network 2) (Figure 1 and Figure A1). A gene signature is required to infer potential master regulators. A common strategy to obtain a gene signature for a given tumor is through differential expression analysis by comparing the cancer cells against the cell of origin. Since the cell of origin of ES is still a matter of debate, we have used two different cell types to infer ES signature: human mesenchymal stem cells (hMSC) and human neural crest cells (hNCC), the most accepted cells of origin in the literature [4]. The two signatures were used to perform the MRA for network 1 and network 2 (Figure A1). Figure 1A,B show tree-and-leaf representations of both regulatory networks (for the regulatory networks with fully regulon names, see Figure A2 and Figure A3). Each network is depicted based on regulon overlap, and the nodes represent the inferred regulons containing at least 15 genes. Networks 1 and 2 contain 645 and 568 regulons, respectively. The MRA performed in the network 1 identified 44 master regulators (Table A1) for the hMSC signature and 13 master regulators for the hNCC signature (Table A2). In the network 2, MRA identified 66 master regulators for the hMSC (Table A3) signature and 42 master regulators for the hNCC signature (Table A4).

Among those four sets of inferred master regulators (MRs), seven are always present: ARNT2, CREB3L1, GLI3, MEF2C, PBX3, PAX7, and RUNX3 (Figure 1A,B) and Figure A1). From that point, we chose to work with those seven master regulators composing the intersection of both networks, and both signatures (Figure A1). In network 1, five master regulators (CREB3L1, GLI3, MEF2C, PBX3, and PAX7) out of the seven common to all MRA analyses are located close to each other at a small region of the network (Figure 1A). In network 2, five of those master regulators (CREB3L1, GLI3, PBX3, RUNX3, and PAX7) are also located close to each other at a small region of the network (Figure 1B).

### 3.2. Differential Methylation Analysis

As a further regulon validation, we investigated the regulon methylation profile in an independent dataset involving ES cell lines and hMSC cell lines. Figure 1C shows that the regulons of the seven identified master regulators (ARNT2, CREB3L1, GLI3, MEF2C, PBX3, PAX7, and RUNX3) have more genes differentially methylated as expected by change in both network 1 and network 2.

### 3.3. Biological Function of Master Regulators Associated Genes

**Figure 2:**
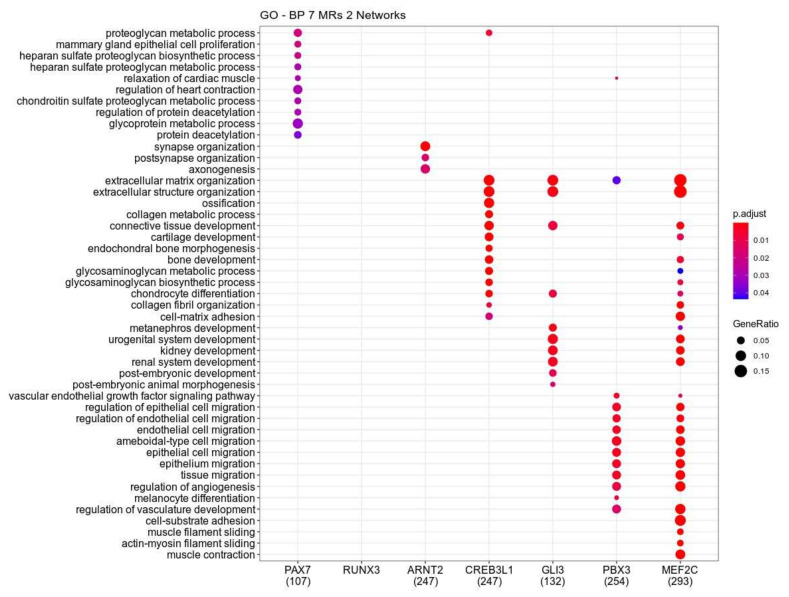
Gene Ontology functional enrichment using clusterProfileR to show the main biological functions performed by each regulon of the seven putative master regulators. The *p*-value cutoff used is 0.05 and the *p*-value was adjusted by Benjamini-Hochberg correction. Each regulon in this analysis consists of the genes regulated by each master regulator in Network 1 and 2.

To perform the functional enrichment of the genes regulated by the seven master regulators (MRs), we considered collectively the genes associated with each MR in both networks 1 and 2. According to Figure 2, PAX7 regulated genes are associated with glycoprotein metabolic process, proteoglycan metabolism, and protein deacetylation. There are no biological functions significantly enriched for RUNX3 regulated genes, while ARNT2 associated genes are involved with synapse and postsynapse organization, and axogenesis. The regulons CREB3L1, GLI3, PBX3, and MEF2C share biological functions, mainly functions involved with extracellular matrix dynamics. For example, the GO term extracellular matrix organization is enriched in those four regulons. GO terms extracellular structure organization, connectivity tissue development, and chondrocyte differentiation are enriched in regulons CREB3L1, GLI3, and MEF2C, while MRs PBX3 and MEF2C regulates genes involved with endothelial and epithelial cell migration.

### 3.4. Regulon Activity

The regulon activity is calculated based on the expression of genes into the regulon. In other words, a regulon is considered to be activated when the genes identified as positively regulated by the transcription factor (in this case, the master regulator) are up-regulated in the GSEA, and the genes negatively regulated by the TF are down-regulated in the GSEA. On the contrary, the regulon is considered inhibited when negatively regulated genes are on the GSEA top, and positively regulated genes are on the GSEA tail. We assessed regulon activity in the different samples used for networks 1 and 2 inference.

**Figure 3:**
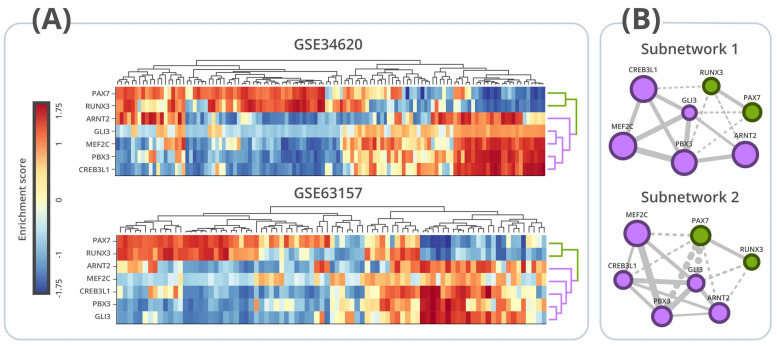
Regulon activity analyses. (**A**) Heatmaps of regulatory activity obtained for both datasets (GSE34620 *N* = 117 and GSE63157 *N* = 85). (**B**) Association map of subnetwork 1 (GSE34620) and subnetwork 2 (GSE63157) involving only the 7 MRs identified in both networks when using the two gene signatures (hMSC and hNCC). Node size reflects the number of regulated genes into regulon, and edge width reflects the number of mutually regulated genes between each regulon. Nodes are colored according to the cluster they belong to. Dotted edges represent regulatory antagonism, while solid edges represent regulatory agonism.

**Figure A4:**
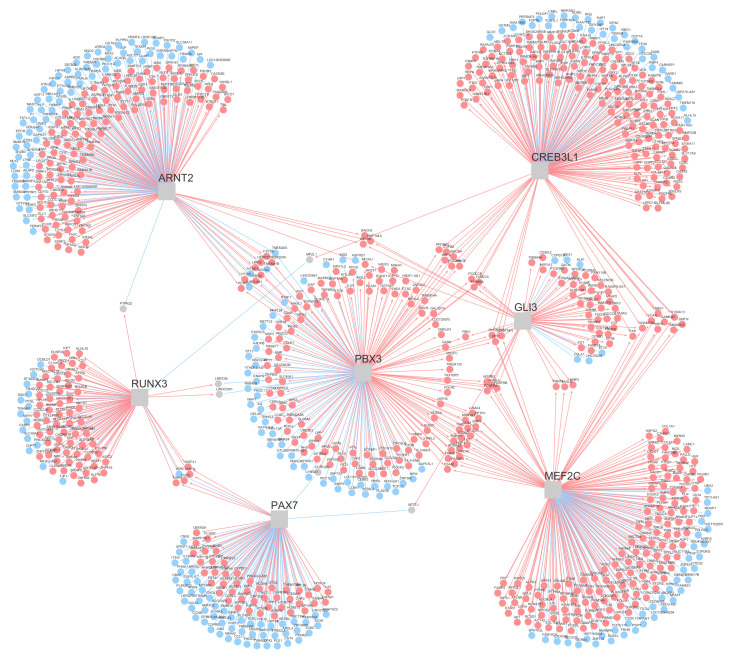
Regulatory map (TF-centric regulatory network) of the seven common master regulators in network 1 (GSE34620). Gray squares are transcription factors (TFs) that are here inferred as mater regulators (MRs) and circles are genes regulated by at least one of the seven MRs. The edges indicate regulation and the color of the edge indicates the type of regulation (red for positive regulation, activation, and blue for negative regulation, inhibition). The circles follow the same color scheme, except that whenever a gene is positively regulated by one TF and negatively regulated by another TF, it is colored gray.

**Figure A5:**
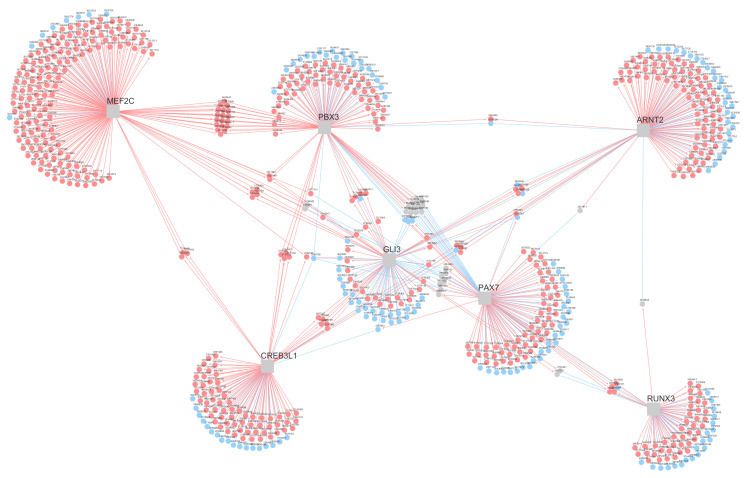
Regulatory map (TF-centric regulatory network) of the seven common master regulators in network 2 (GSE63157). Gray squares are transcription factors (TFs) that are here inferred as mater regulators (MRs) and circles are genes regulated by at least one of the seven MRs. The edges indicate regulation and the color of the edge indicates the type of regulation (red for positive regulation, activation, and blue for negative regulation, inhibition). The circles follow the same color scheme, except that whenever a gene is positively regulated by one TF and negatively regulated by another TF, it is colored gray.

Figure 3A shows the heatmaps involving the activity of the seven MR for the 117 samples used to infer network 1 (GSE34620) and the 85 samples used to infer network 2 (GSE63157). In both heatmaps, it is possible to observe two clusters: one formed by regulons PAX7 and RUNX3 and another formed by regulons ARNT2, CREB3L1, GLI3, MEF2C, and PBX3. The regulatory activity of these two clusters is antagonistic to each other in both cohorts: in general, patients with high RUNX3 and PAX7 regulon activity have low ARNT2, CREB3L1, GLI3, MEF2C, and PBX3 regulon activity and vice-versa (Figure 3A). Figure 3B shows two subnetworks extracted from the regulatory networks 1 and 2. Both subnetworks include only the seven regulons used for heatmap analyses (Figure 3A). Similar to Figure 1, the network is depicted according to regulon overlapping (i.e., according to genes mutually regulated), but here the edges indicate whether each pair of transcription factors regulates the shared genes in the same direction (agonistic regulation) or in the opposite direction (antagonistic regulation). As seen in Figure 3B, it is possible to observe two groups of regulons. PAX7 and RUNX3 regulate shared genes in the same direction. Similarly, the group formed by ARNT2, CREB3L1, GLI3, MEF2C, and PBX3 regulates the shared genes in the same direction (Figure 3A and Figure A4 and Figure A5). However, transcription factors of different groups always regulate simultaneously regulated genes in the opposite direction (Figure 3B). According to Figure 3, regulons PAX7 and RUNX3 are simultaneously activated or inhibited and act coordinately by regulating shared genes in the same direction. The same occurs among regulons ARNT2, CREB3L1, GLI3, MEF2C, and PBX3. Moreover, both groups seem to work as antagonists between each other.

### 3.5. Survival Analysis

**Figure 4:**
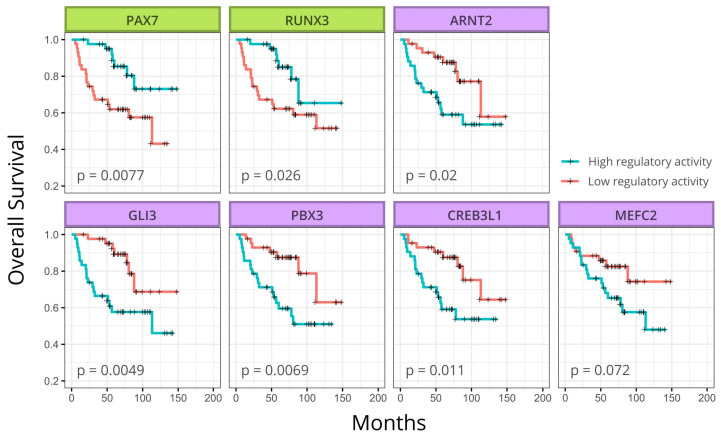
Kaplan-Meier plot of ES patients. High regulatory activity of PAX7 and RUNX3 is associated with better patient overall survival, while high regulatory activity of ARNT2, GLI3, PBX3, and CREB3L1 is associated with worse overall survival. Box colors reflect the cluster each regulon belongs to. *p*-values are presented inside each box. Survival and expression data were obtained from ES dataset GSE63157 (N = 85).

To evaluate the impact of the seven regulon activity in ES outcome, we accessed survival data available for the 85 patients from the cohort used to infer ES regulatory network 2 (GSE63157). The samples into the cohort were classified according to the activity of each regulon. Figure 4 shows the seven Kaplan-Meier plots corresponding to each regulon where six of them were significantly related to patient outcome. Again, PAX7 and RUNX3 present similar behavior regarding patient outcome with both regulons associated with a good prognosis when activated. In contrast, regulons ARNT2, CREB3L1, GLI3, and PBX3 are associated with bad prognosis when activated.

**Figure 5:**
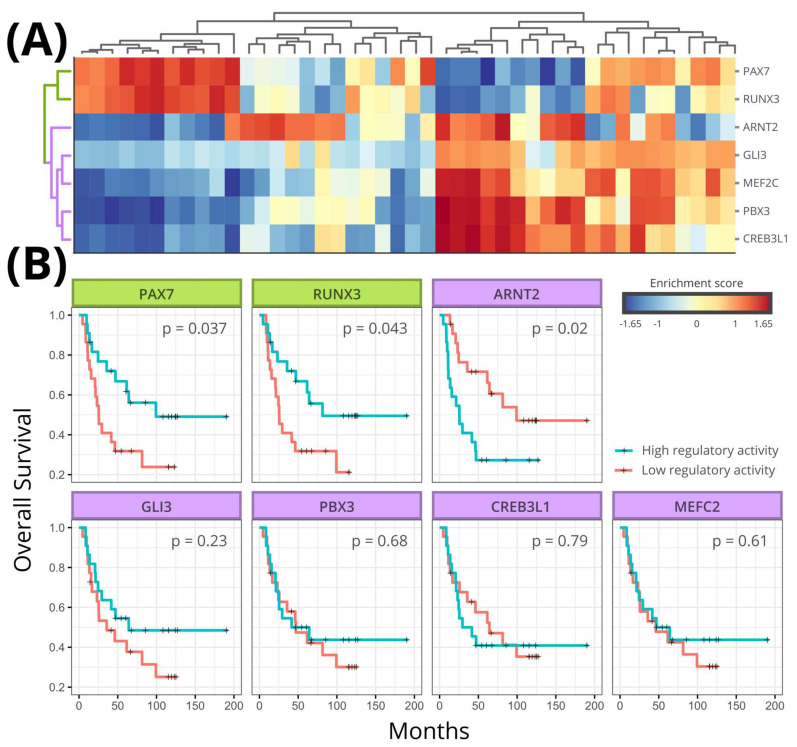
Regulatory activity and survival analyses (ES dataset GSE17618, *N* = 44). (**A**) Heatmaps of regulatory activity (**B**) Kaplan Meier plots obtained with survival and gene expression data obtained from GSE17618. High regulatory activity of PAX7 and RUNX3 regulons is associated with better overall survival, while high regulatory activity of ARTN2 is associated with worse overall survival. Box colors reflect the cluster each regulon belongs to. *p*-values are presented inside each box.

Unfortunately, there is no survival data available regarding patients from cohort 1 (GSE34620). To verify the implication of regulon activity in patient outcome, we access another ES cohort composed by 44 patients (GSE17618). We evaluated the activity of the seven regulons inferred for network 1 in each of 44 patients with data available in GSE17618. Figure 5A shows the heatmap clustering based on regulon activity. The heatmap presents the same pattern observed in Figure 3A. PAX7 and RUNX3 regulons cluster together, in contrast to regulons ARNT2, CREB3L1, GLI3, and PBX3. Additionally, both PAX7 and RUNX3 are significantly related to good prognosis when activated. Among the other regulons, only ARNT2 was significantly related to patient outcome, being associated with bad prognosis when activated.

### 3.6. Master Regulators Expression in Ewing Sarcoma

**Figure 6:**
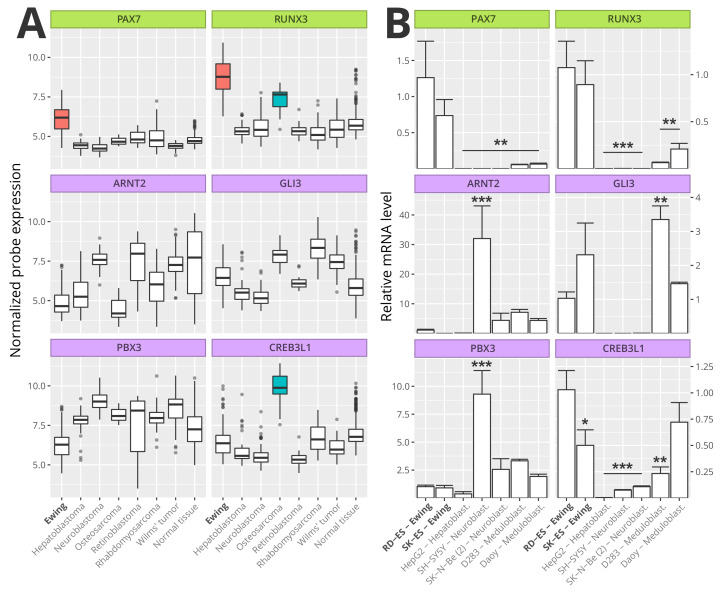
Ewing Sarcoma Master Regulators Expression. The boxplots (**A**) show the expression of the six ES master regulators significantly associated with patient outcome (PAX7, RUNX3, ARNT2, GLI3, PBX3, and CREB3L1). The expression of each MRs was assessed in biopsies from ES (N = 117), Hepatoblastoma (N = 55), Neuroblastoma (N = 88), Osteosarcoma (N = 21), Retinoblastoma (N = 20), Rhabdomyosarcoma (N = 58), Wilm’s tumor (N = 53) and a dataset from 65 different healthy tissues as control (N = 353). Colored boxes indicate the group was significantly different compared to all the other groups (Pairwise Wilcox test with Bonferroni correction, *p* < 0.05). Bar plots (**B**) represent the relative expression measured by RT-qPCR of the indicated MRs in different cell lines originated from ES and other pediatric tumors. The differences are always related to RD-ES cell line (* *p* < 0.05, ** *p* < 0.01, *** *p* < 0.0001).

We evaluated the expression of ES master regulators significantly associated with patient outcome (i.e., PAX7, RUNX3, ARNT2, GLI3, PBX3, and CREB3L1) in ES samples as well as in samples of the most common solid pediatric tumors: neuroblastoma, Wilm’s tumor, hepatoblastoma, osteosarcoma, retinoblastoma, and rhabdomyosarcoma [36]. We also evaluated the expression of the above master regulators in a set of samples (N = 353) from 65 different healthy tissues (Figure 6). PAX7 and RUNX3 genes were highly expressed in ES samples when compared with the other evaluated pediatric tumors and normal tissues. RUNX3 was also highly expressed in osteosarcoma when compared with normal tissue, neuroblastoma, Wilm’s tumor, hepatoblastoma, retinoblastoma, and rhabdomyosarcoma. However, RUNX3 expression in ES was significantly higher when compared with the expression in osteosarcoma. The expression of the other four master regulators (ARNT2, GLI3, PBX3, and CREB3L1) had no significant difference among the samples, except by CREB3L1 gene which was highly expressed in osteosarcoma samples (Pairwise Wilcox test with Bonferroni correction, *p*-value < 0.01).

In sense to validate the transcriptome data, the transcript levels of ES master regulators genes were evaluated in representative cell lines of ES, neuroblastoma, hepatoblastoma and medulloblastoma (Figure 6B). The expression of PAX7 and RUNX3 were similar between ES cell lines and were significantly higher in RD-ES cell line in comparison to other tumor cell lines (*p* < 0.01). ARNT2 and PBX3 expressions were lower in ES cell line RD-ES than neuroblastoma cell line SH-5Y5Y (*p* < 0.001). GLI3 was highly expressed in medulloblastoma cell line D283 compared to RD-ES (*p* < 0.01). CREB3L1 expression was higher in the ES cell line RD-ES compared to neuroblastoma, hepatoblastoma (*p* < 0.001) and D283 cell line (*p* < 0.01), whereas no significant difference was observed in Daoy cells.

## 4. Discussion

The primary goal of this study was to reconstruct the ES regulatory network to understand the ES regulatory properties and, particularly, to identify transcription factors potentially relevant to that cancer. However, the uncertainty regarding ES cell of origin hinders the identification of master regulators. To surpass that limitation, we inferred two signatures using the two most accepted ES cells of origin: hMSC and hNCC [4]. The number of inferred master regulators vary according to the used signatures, but the master regulators ARNT2, CREB3L1, GLI3, MEF2C, PBX3, PAX7, and RUNX3 were found using either signature in both networks. It would be naive to consider only those seven TFs as Ewing Sarcoma regulators. It is reasonable to assume that other TFs identified as master regulators only when using hMSC signature could be relevant to ES transcriptional regulation, especially if hMSC were the ES cell of origin. The same is true for the hNCC signature. As example, the transcription factors NR0B1 and NKX2-2 are well-known to be associated with Ewing Sarcoma [37]. NR0B1 have been associated with the tumor phenotype mediated by EWS/FLI chimera in cell lines [38]. NKX2–2, a homeodomain transcription factor involved in neuroendocrine/glial differentiation and a downstream target of EWSR1-FLI1, has been reported as an immunohistochemical marker for Ewing sarcoma [39]. We identified both NR0B1 and NKX2-2 as MRs in our analysis. NR0B1 was identified as MR in network 2 using both hMSC and hNCC signatures (Table A3 and Table A4, and Figure A2 and Figure A3), while NKX2-2 was identified in network 1 using hNCC signature (Table A2 and Figure A2), and in network 2 using the two signatures (Table A3 and Table A4, and Figure A3). Because they were not always present in the four master regulator analysis (i.e., networks 1 and 2, using both hMSC and hNCC signatures), they were left out of the further analysis. Another sensitive point is the Ewing Sarcoma cell of origin. Despite hMSC and hNCC being the most supported cells of origin by the literature [2,10,40,41,42,43,44], we can not neglect the possibility of other cells of origin. However, our stringent strategy assures that the seven master regulators inferred here are involved in the ES transcriptional regulation.

According to our results, the regulons ARNT2, CREB3L1, GLI3, MEF2C, and PBX3 act coordinately: (i) they are collectively activated or collectively inhibited in the majority of the patients from the three cohorts evaluated here; (ii) those five regulons always regulate shared genes in the same direction; (iii) except for MEF2C, all of those regulons are significantly related with poor prognosis in cohort GSE63157 (N = 85) when activated. The same statement is true for PAX7 and RUNX3, except that they are both associated with good prognosis when activated. We also evaluated overall survival of a small cohort (GSE17618) with 44 patients with similar results: PAX7 and RUNX3 are significantly associated with good prognosis when activated, and ARNT2 is significantly associated with poor prognosis when activated. Interestingly, both clusters—the first formed by ARNT2, CREB3L1, GLI3, MEF2C, and PBX3, and the second formed by PAX7 and RUNX3—act collectively as reciprocal antagonists to each other regarding activation, regulation of shared genes, and implication in patient outcome. A similar behavior can be observed in the biological functions performed by the regulons. For instance, CREB3L1, GLI3, PBX3, and MEF2C share biological functions involved with extracellular matrix dynamics, and all those regulons are agonist among each other and cluster together.

The results of our methylation analysis show that all the seven regulons discussed in the manuscript are differentially methylated in an independent set of ES samples, reinforcing our findings and highlighting the importance of epigenomic reprogramming in the tumour regulation, as demonstrated by Sheffield et al that pointed out epigenetic heterogeneity in genetically homogeneous developmental cancers [45]. However, it was not possible to associate the methylation experiment with the regulon activity since we do not have the same set of samples with both methylation and expression experiments.

It is not clear why some patients have the cluster composed of PAX7 and RUNX3 regulons activated, while the same cluster is inhibited in other patients, and further investigations are needed to elucidate it. However, the differential regulon activity among patients seems to be important to tumor prognostic since we showed it is associated with overall survival. Similar result was observed in breast cancer, where it was possible to stratify a patient cohort based on regulon activity [13].In the same work, the authors have shown that the pharmacological inhibition of estrogen receptor drastically suppresses the ESR1 regulon. The ESR1 regulon has either estrogen-induced and estrogen-repressed genes. Therefore, their function as regulator occurs oppositely when the regulon is repressed and when the regulon is activated [13]. It suggests that the MR influence can be through the activation of its targets or by target inhibition, and both activation and repression of an MR might modulate the cell fate.

In other studies, ARNT2, CREB3L1, GLI3, and PBX3 have been associated with tumor progression. The role of CREB3L1 in tumor phenotype is controversial: while some studies associated this TF with metastasis promotion, other studies suggest its role in metastasis inhibition [46,47]. ARNT2 was pointed out as a key TF in the control of glioblastoma cell aggressiveness by regulating the expression of TFs related to a tumorigenic/stem glioblastoma signature [48]. PBX3 is well described as associated with poor prognostic in several cancer types [49], and the same is correct for GLI3 [50,51]. However, the role of those TFs has not been previously reported in ES. Previous studies have suggested that high expression of CREB3L1, GLI3, MEF2C, and PBX3 induces invasion and metastasis by promoting epithelial–mesenchymal transition (EMT) [47,51,52], a process reported as critical to induce metastasis in ES [53]. ARTN2 is associated with hypoxia response and acts as a dimerization partner of hypoxia-inducible factor 1α (HIF1α), which acts is adaptive stress response as well as angiogenesis required for tumor growth and metastasis [54,55].

Functional enrichment analysis has shown ARNT2, CREB3L1, GLI3, and PBX3 regulons related to several processes associated with cell migration such as extracellular matrix organization, collagen metabolic process, glycosaminoglycan (GAG) process, epithelial cell migration, and regulation of angiogenesis. Cell migration is an essential process for regulating cancer invasion. An initial step in cancer metastasis is the migration of tumor cells through the extracellular matrix (ECM) and into the lymphatic or vascular systems [56]. Progression to metastasis is associated with some biomechanical particularities, such as the restructuring of the extracellular matrix, collagen organization, ECM environments rich in the GAG, angiogenesis process, and epithelial cell migration [57,58,59,60] all process regulated by the ARNT2, CREB3L1, GLI3, and PBX3 regulons according to our results. Therefore, the high activity of ARNT2, CREB3L1, GLI3, and PBX3 regulons could be related to ES aggressiveness through stress adaptation, angiogenesis, and mesenchymal-like phenotype induction [61].

Recent studies suggested that EWSR1-FLI1 chimera regulates the expression of PAX7 and RUNX3 [62,63]. According to the available evidence, PAX7 is required for neural crest formation and adult skeletal muscle progenitor development [64]. PAX7 is described to be expressed in ES, subsets of rhabdomyosarcoma, and rare synovial sarcomas [65,66]. Baldauf et al 2018, interrogated Chaville’s findings, since the increased expression of the PAX7 gene was observed in a dataset that compares samples of CIC-DUX4-positive sarcomas with EWSR1-NFATc2-positive sarcomas. According Baldauf et al. 2018, EWSR1-NFATC2-positive sarcomas are transcriptionally distinct from tumors with EWSR1-FLI1 translocation and should be treated as an entity distinct from EWSR1-ETS tumors [67]. On the other hand, Toki et al. 2018 using monoclonal antibody against PAX7 identified the PAX7 expression in 27 of 30 molecularly confirmed Ewing Sarcomas (90%) [66]. However, its role in tumor biology is uncertain. Charville and collaborators suggested that EWSR1-FLI1 binds to the PAX7 promoter, being the chimeric protein required for PAX7 expression in ES [62]. The transcription factor RUNX3 has been described as a tumor suppressor [68]. Bledsoe and collaborators verified that RUNX3 is expressed in ES cell lines as well as in tumor biopsies. Additionally, RUNX3 inhibition alters the expression of a set of genes regulated by EWSR1-FLI1 in A673 cell line. The authors also observed that suppression of RUNX3 expression in A673 reduced cell growth [63]. On the other hand, several studies demonstrated RUNX3 inhibition in different cancers, such as colorectal cancer, glioma, melanoma, and breast cancer [69,70,71,72].

The opposite behavior of PAX7 and RUNX3 when compared with ARNT2, CREB3L1, GLI3, and PBX3 suggests that the first two TFs could act by antagonizing the last four TFs action. As mentioned before, ARNT2, CREB3L1, GLI3, and PBX3 are well described in several tumors, while PAX7 and RUNX3 seem to be more specific to ES. When taken together, our results allow us to hypothesize that PAX7 and RUNX3 activation in ES could help mitigate the damaging effect caused by ARNT2, CREB3L1, GLI3, and PBX3 activation. PAX7 is known to be expressed in ES, subsets of rhabdomyosarcoma, and rare cases of synovial sarcomas, though only in ES samples it was found positive in all evaluated cases [65]. Charville and collaborators suggest that significant expression of PAX7 is a unique feature of rhabdomyosarcoma and ES, and put forward PAX7 as a diagnostic marker for ES diagnosis [62]. Additionally, we also identified RUNX3 as highly expressed in ES, corroborating with the similar regulatory behavior shared by those two transcription factors in ES regulatory network. High expression of PAX7 and RUNX3 genes could help mitigate EMT promoted by the cluster formed by ARNT2, CREB3L1, GLI3, and PBX3, contributing to avoid metastasis and therefore an aggressive behavior that often leads to death. Even though there is no evidence that PAX7 and RUNX3 promote mesenchymal-epithelial-transition (MET), which is the opposite of EMT, they may be able to avoid metastasis only by avoiding EMT.

## 5. Conclusions

The regulatory network analysis sheds light on the Ewing Sarcoma regulatory behavior by identifying PAX7 and RUNX3 as promising master regulators for this cancer. Both regulons are agonists, are simultaneously activated or inhibited in patient samples, and are both associated with a good prognosis. The analysis evinces another cluster of regulon consisting of ARNT2, CREB3L1, GLI3, MEF2C, and PBX3, which counteracts PAX7 and RUNX3 in all the parameters mentioned above, suggesting that the last two regulons counteract the former five regulons. 

## Data Availability

MicroArray and RNA sequenced samples mentioned in this research can be accessed at the Gene Expression Omnibus (https://www.ncbi.nlm.nih.gov/geo/ (accessed on: 22 February 2021)) with the accession numbers: GSE34620, GSE63157, GSE73610, GSE67073, GSE17618, GSE16476, GSE53224, GSE75271, GSE87437, GSE29684, GSE66533, and GSE3526. Source code of the analyses mentioned in this study can be found at https://github.com/dalmolingroup/ewing-mra-netinfer.

## Abbreviations

The following abbreviations are used in this manuscript:

2GSEA	Two-Tailed Gene Set Enrichment Analysis
ANOVA	Analysis of Variance
ARACNE	Algorithm for the Reconstruction of Accurate Cellular Networks
CHTN	Cooperative Human Tissue Network
CIT	Cartes d’Identité des Tumeurs research program
COG	Children’s Oncology Group
EICESS	European Intergroup Cooperative Ewing’s Sarcoma Study
ES	Ewing Sarcoma
GSEA	Gene Set Enrichment Analysis
GSE	GEO Series
GSM	GEO Sample
hMSC	Human Mesenchymal Stem Cells
hNCC	Human Neural Crest Cells
iPSC	Induced Pluripotent Stem Cells
MR	Master Regulator
MRA	Master Regulator Analysis
RT-PCR	Real-time polymerase chain reaction
TF	Transcription Factor

## Appendix B

**Table A1:** This table contains the 44 master regulators (MRs) inferred for network 1 with the signature obtained with human Mesenchymal Stem Cells. *p*-values were adjusted with Benjamini & Hochberg—BH.

MR	*p*-Value	Adjusted *p*-Value
CREB3L1	1.80 × 10${}^{-11}$	1.20 × 10${}^{-8}$
AEBP1	4.80 × 10${}^{-9}$	1.50 × 10${}^{-6}$
SNAI2	8.00 × 10${}^{-9}$	1.70 × 10${}^{-6}$
PBX3	1.10 × 10${}^{-8}$	1.80 × 10${}^{-6}$
SMAD7	6.80 × 10${}^{-8}$	8.70 × 10${}^{-6}$
NR6A1	2.10 × 10${}^{-7}$	2.30 × 10${}^{-5}$
SOX11	5.10 × 10${}^{-7}$	4.30 × 10${}^{-5}$
HIC1	5.40 × 10${}^{-7}$	4.30 × 10${}^{-5}$
AHR	8.50 × 10${}^{-7}$	6.10 × 10${}^{-5}$
CTBP2	1.40 × 10${}^{-6}$	8.90 × 10${}^{-5}$
TFAP2B	2.60 × 10${}^{-5}$	0.0009
HHEX	2.70 × 10${}^{-5}$	0.0009
TRPS1	3.70 × 10${}^{-6}$	0.00022
HIF1A	6.00 × 10${}^{-6}$	0.00027
HOXB13	5.50 × 10${}^{-6}$	0.00027
SHOX2	5.50 × 10${}^{-6}$	0.00027
EPAS1	7.80 × 10${}^{-6}$	0.00033
KLF9	2.10 × 10${}^{-5}$	0.00083
ELK3	2.20 × 10${}^{-5}$	0.00083
KLF10	3.20 × 10${}^{-5}$	0.001
IRF2	3.30 × 10${}^{-5}$	0.001
FOSL2	3.80 × 10${}^{-5}$	0.0011
PAX7	6.90 × 10${}^{-5}$	0.0018
XBP1	6.90 × 10${}^{-5}$	0.0018
KLF11	6.70 × 10${}^{-5}$	0.0018
MEIS1	0.00012	0.0029
CREG1	0.00012	0.003
IRF7	0.00029	0.0067
ELF4	0.00038	0.0084
SIX2	0.00043	0.0093
RUNX3	0.0008	0.016
ESR1	0.00076	0.016
ZNF667	0.00094	0.018
MEF2C	0.0013	0.025
GAS7	0.0015	0.028
ZNF124	0.0016	0.029
CBFB	0.0017	0.03
GATA6	0.0017	0.03
GLI3	0.0019	0.032
RUNX2	0.0021	0.035
ARNT2	0.0026	0.042
ZNF34	0.0028	0.043
ETS1	0.003	0.045
ZNF195	0.0031	0.045

**Table A2:** This table contains the 13 MRs inferred for network 1 with the signature obtained with human Neural Crest Cells. *p*-values were adjusted with Benjamini & Hochberg—BH.

MR	*p*-Value	Adjusted *p*-Value
PAX7	5.00 × 10${}^{-5}$	6.50 × 10${}^{-3}$
AEBP1	1.50 × 10${}^{-4}$	1.40 × 10${}^{-2}$
ARNT2	8.20 × 10${}^{-6}$	3.80 × 10${}^{-3}$
PBX3	1.70 × 10${}^{-5}$	3.80 × 10${}^{-3}$
RUNX3	5.30 × 10${}^{-4}$	3.40 × 10${}^{-2}$
NKX2-2	2.20 × 10${}^{-5}$	3.80 × 10${}^{-3}$
MEF2C	2.40 × 10${}^{-5}$	3.80 × 10${}^{-3}$
NOTCH2	1.90 × 10${}^{-4}$	1.50 × 10${}^{-2}$
CREB3L1	1.30 × 10${}^{-4}$	1.40 × 10${}^{-2}$
ZNF423	6.80 × 10${}^{-4}$	3.70 × 10${}^{-2}$
TEF	6.00 × 10${}^{-4}$	0.035
GLI3	9.70 × 10${}^{-4}$	0.048
SOX1	4.70 × 10${}^{-4}$	0.034

**Table A3:** This table contains the 66 master regulators (MRs) inferred for network 2 with the signature obtained with human Mesenchymal Stem Cells. *p*-values were adjusted with Benjamini & Hochberg—BH.

MR	*p*-Value	Adjusted *p*-Value
SNAI2	8.30 × 10${}^{-37}$	4.70 × 10${}^{-34}$
MEF2C	1.20 × 10${}^{-22}$	3.40 × 10${}^{-20}$
PAX7	5.00 × 10${}^{-17}$	9.50 × 10${}^{-15}$
RUNX1	1.40 × 10${}^{-16}$	2.00 × 10${}^{-14}$
CREB3L1	1.00 × 10${}^{-15}$	1.20 × 10${}^{-13}$
AEBP1	3.30 × 10${}^{-14}$	3.10 × 10${}^{-12}$
NKX2-2	9.00 × 10${}^{-14}$	7.30 × 10${}^{-12}$
PBX3	1.20 × 10${}^{-12}$	8.20 × 10${}^{-11}$
AHR	2.50 × 10${}^{-12}$	1.60 × 10${}^{-10}$
CTNNB1	6.50 × 10${}^{-12}$	3.70 × 10${}^{-10}$
XBP1	8.50 × 10${}^{-12}$	4.40 × 10${}^{-10}$
EPAS1	3.50 × 10${}^{-10}$	1.70 × 10${}^{-8}$
BACH2	1.30 × 10${}^{-9}$	5.60 × 10${}^{-8}$
GLI3	1.40 × 10${}^{-9}$	5.60 × 10${}^{-8}$
SOX11	1.80 × 10${}^{-9}$	6.90 × 10${}^{-8}$
ETV1	1.30 × 10${}^{-8}$	4.20 × 10${}^{-7}$
MAF	1.20 × 10${}^{-8}$	4.20 × 10${}^{-7}$
ETS1	1.60 × 10${}^{-8}$	4.90 × 10${}^{-7}$
TCF4	2.70 × 10${}^{-8}$	8.20 × 10${}^{-7}$
CREG1	3.60 × 10${}^{-8}$	1.00 × 10${}^{-6}$
MEOX2	5.20 × 10${}^{-8}$	1.40 × 10${}^{-6}$
RUNX3	1.90 × 10${}^{-7}$	4.80 × 10${}^{-6}$
ETV5	2.20 × 10${}^{-7}$	5.40 × 10${}^{-6}$
TRPS1	8.80 × 10${}^{-7}$	2.10 × 10${}^{-5}$
FOSL2	1.50 × 10${}^{-6}$	3.30 × 10${}^{-5}$
HEY2	1.70 × 10${}^{-6}$	3.70 × 10${}^{-5}$
CEBPB	7.30 × 10${}^{-6}$	0.00015
PRDM1	9.40 × 10${}^{-6}$	0.00019
PKNOX2	1.20 × 10${}^{-5}$	0.00023
ZNF74	1.30 × 10${}^{-5}$	0.00024
ARNTL2	1.80 × 10${}^{-5}$	0.00033
SOX1	2.00 × 10${}^{-5}$	0.00035
IRF2	2.30 × 10${}^{-5}$	0.00039
ETV4	4.50 × 10${}^{-5}$	0.00075
ELF1	5.30 × 10${}^{-5}$	0.00086
ZBTB25	7.40 × 10${}^{-5}$	0.0011
ARNT2	7.60 × 10${}^{-5}$	0.0011
NR0B1	7.50 × 10${}^{-5}$	0.0011
RUNX2	8.00 × 10${}^{-5}$	0.0012
ESRRB	0.00016	0.0023
ZBTB16	0.00017	0.0024
KLF9	0.0002	0.0027
TFEC	0.00021	0.0028
HEYL	0.00028	0.0036
HCLS1	0.00031	0.0039
HR	0.0005	0.0062
MEIS2	0.00064	0.0076
ZFP36L2	0.0007	0.0082
KLF4	0.00078	0.009
CREB3L2	0.00088	0.01
HIVEP1	0.0012	0.013
ZNF516	0.0012	0.013
ZNF235	0.0013	0.014
MYT1	0.0015	0.016
ZNF219	0.0016	0.017
AFF3	0.0021	0.021
HIVEP3	0.0024	0.024
CEBPD	0.0027	0.026
TEAD1	0.0028	0.027
SMAD5	0.0031	0.029
NR3C1	0.0041	0.038
NFE2L1	0.0043	0.039
NKX6-1	0.0049	0.043
ZNF550	0.0049	0.043
STAT1	0.0054	0.047
SATB2	0.0058	0.05

**Table A4:** This table contains the 42 MRs inferred for network 2 with the signature obtained with human Neural Crest Cells. *p*-values were adjusted with Benjamini & Hochberg—BH.

MR	*p*-Value	Adjusted *p*-Value
PBX3	1.80 × 10${}^{-13}$	1.00 × 10${}^{-10}$
PKNOX2	3.90 × 10${}^{-13}$	1.10 × 10${}^{-10}$
PAX7	3.30 × 10${}^{-12}$	6.20 × 10${}^{-10}$
CEBPB	1.60 × 10${}^{-11}$	2.20 × 10${}^{-9}$
GLI3	2.30 × 10${}^{-9}$	2.60 × 10${}^{-7}$
AFF3	9.20 × 10${}^{-9}$	7.40 × 10${}^{-7}$
ZNF215	9.20 × 10${}^{-9}$	7.40 × 10${}^{-7}$
MYT1	1.20 × 10${}^{-8}$	7.60 × 10${}^{-7}$
RUNX3	1.20 × 10${}^{-8}$	7.60 × 10${}^{-7}$
ARNT2	5.00 × 10${}^{-8}$	2.80 × 10${}^{-6}$
ETV4	1.40 × 10${}^{-7}$	7.20 × 10${}^{-6}$
CREB3L1	6.30 × 10${}^{-7}$	3.00 × 10${}^{-5}$
MYT1L	1.60 × 10${}^{-6}$	7.00 × 10${}^{-5}$
ZBTB25	1.80 × 10${}^{-6}$	7.40 × 10${}^{-5}$
MEOX2	4.30 × 10${}^{-6}$	0.00016
SNAI2	8.70 × 10${}^{-6}$	0.00031
RUNX1	9.40 × 10${}^{-6}$	0.00031
NKX2-2	1.60 × 10${}^{-5}$	0.00051
MSC	3.50 × 10${}^{-5}$	0.001
ETV1	1.10 × 10${}^{-4}$	0.0031
MAF	1.10 × 10${}^{-4}$	0.0031
MYC	1.40 × 10${}^{-4}$	0.0036
HOXB9	1.60 × 10${}^{-4}$	0.0039
MEF2C	1.80 × 10${}^{-4}$	0.0044
LMO1	2.40 × 10${}^{-4}$	0.0055
ETS1	0.00029	0.0063
MEIS2	0.00041	0.0086
SOX17	0.00068	0.014
BACH2	0.00069	0.014
HEY2	0.00076	0.014
HIVEP3	0.00097	0.018
ARNTL2	0.001	0.018
SMAD9	0.001	0.018
TCF4	0.0011	0.019
HOXC11	0.0014	0.023
NR0B1	0.0019	0.029
ESRRB	0.0022	0.033
PRDM1	0.0024	0.035
SMAD6	0.0026	0.037
NR2F1	0.003	0.042
SALL1	0.0036	0.049
TCF7L2	0.0037	0.05
